# RIPK1 inhibitor ameliorates pulmonary injury by modulating the function of neutrophils and vascular endothelial cells

**DOI:** 10.1038/s41420-024-01921-8

**Published:** 2024-03-23

**Authors:** Tao Yang, Cai-gui Xiang, Xiao-han Wang, Qing-qing Li, Shu-yue Lei, Kai-rong Zhang, Jing Ren, Hui-min Lu, Chun-lan Feng, Wei Tang

**Affiliations:** 1 State Key Laboratory of Chemical Biology, Shanghai Institute of Materia Medica, Chinese Academy of Sciences, Shanghai, 201203 China; 2 School of Pharmacy, University of Chinese Academy of Sciences, Beijing, 100049 China; 3 School of Pharmaceutical Science, Nanchang University, Nanchang, 330006 China; 4 School of Chinese Materia Medica, Nanjing University of Chinese Medicine, Nanjing, 210000 China

**Keywords:** Acute inflammation, Cytokines

## Abstract

Acute lung injury (ALI) is an acute and progressive hypoxic respiratory failure that could progress to acute respiratory distress syndrome (ARDS) with a high mortality rate, thus immediate medical attention and supportive care are necessary. The pathophysiology of ALI is characterized by the disruption of the alveolar-capillary barrier and activation of neutrophils, leading to lung tissue damage. The receptor-interacting protein kinase 1 (RIPK1) has emerged as a promising target for the treatment of multiple inflammatory diseases, but the role of RIPK1 in the ALI remains poorly understood. In this study, we aimed to figure out the pathological role of RIPK1 in ALI, especially in the pulmonary immune microenvironment involving neutrophils and endothelial cells. In vivo experiments showed that RIPK1 inhibitor protected against lipopolysaccharide (LPS)-induced lung injury in mouse models, with reduced neutrophils and monocytes infiltration in the lungs. Further studies demonstrated that, besides the inhibitory action on necroptosis, RIPK1 inhibitor directly suppressed reactive oxygen species (ROS) generation and inflammatory cytokines secretion from neutrophils. Furthermore, RIPK1 inhibition maintains the barrier function in TNF-α-primed vascular endothelial cells and prevents their activation induced by the supernatant from LPS-stimulated neutrophils. Mechanistically, the aforementioned effects of RIPK1 inhibitor are associated with the NF-κB signaling pathway, which is partially independent of necroptosis inhibition. These results provide new evidence that RIPK1 inhibitor directly regulates the function of neutrophils and endothelial cells, as well as interferes with the interactions between these two cell types, therefore contributing to a better understanding of RIPK1 in ALI and providing a potential avenue for future therapeutic interventions.

## Introduction

Acute lung injury (ALI), a highly prevalent and life-threatening condition, is characterized by acute inflammatory lung injury induced by various direct or indirect factors, such as trauma, infection, inhalation of toxic gases, and septic shock [1]. The development of ALI is attributed to a significant inflammatory response that leads to augmented capillary permeability, subsequent fluid accumulation, disruption of pulmonary gas exchange, and eventually fibrosis [2]. It is important to note that currently, there are no specific medications proven to improve short-term or long-term survival rates in patients with ALI [3]. These challenges have recently gained global attention, particularly due to the ongoing SARS-CoV-2 pandemic.

Receptor-interacting protein kinase 1 (RIPK1) is a crucial downstream regulator of TNF receptor 1 (TNFR) [4]. Its kinase-dependent function triggers necroptosis, a form of regulated necrotic cell death, through the formation of the RIPK1-RIPK3-mixed lineage kinase domain-like protein (MLKL) complex [5]. Necroptosis is defined as an inflammatory form of cell death characterized by the release of a large amount of damage-associated molecular patterns (DAMPs), which subsequently trigger a robust immune response [4]. RIPK1 has been reported to be involved in the progression of various diseases, including colitis, psoriasis, sepsis, viral infections, neurodegenerative diseases, graft vs host disease, lung injury, and atherosclerosis [6]. Given the remarkable anti-inflammatory properties of RIPK1 inhibitors, their potential as a therapeutic target for inflammatory diseases is significantly highlighted [7]. Multiple RIPK1 inhibitors, including GSK2982772 [8], DNL747 [9], DNL-758 (CTR20231473) and AC-003 (CXHL2300230), have been investigated in clinical studies to date. Whereas, the pathological role of RIPK1 and the efficacy of RIPK1 inhibition in acute lung injury need to be fully understood.

The pathogenesis of ALI involves several cellular events, including damage to alveolar epithelial cells, massive infiltration of neutrophils, injury to vascular endothelial cells, and activation of alveolar macrophages [10–13]. Neutrophils are recognized as a significant biomarker of ALI and their presence in patients’ bronchoalveolar lavage fluid (BALF) is directly linked to the severity of the disease [11]. Upon activation, neutrophils discharge an array of toxic substances including reactive oxygen species (ROS), cytokines among others [14]. Similarly, endothelial cells act as a conduit for immune cells, execute a vital paracrine role by expressing adhesion molecules and chemokines to facilitate the recruitment of immune cells [15]. Several studies have indicated that RIPK1 plays a significant role in various types of lung injury. Guan et al. discovered that Necrostatin-1, a RIPK1 inhibitor, can alleviate sepsis-induced lung injury by suppressing inflammatory responses and NF-κB activation in RAW 264.7 cells [16]. Lin et al. found that Necrostatin-1 can relieve LPS-induced lung injury by inhibiting necroptosis of pulmonary epithelial cells and oxidative stress [17]. Dong et al. and Wang et al. discovered that Necrostatin-1 shows promise in treating lung injury caused by pulmonary ischemia-reperfusion by inhibiting the death of pulmonary epithelial cells [18, 19]. However, despite these findings, the influence of RIPK1 signaling in neutrophils and vascular endothelial cells during ALI remains unclear. Moreover, beyond the necroptosis modulation, recent studies have revealed that the activation of RIPK1 can induce inflammation signaling pathways independently of cell death, although the precise molecular mechanisms remain uncertain. For instance, co-treatment of LPS and Z-VAD-FMK upregulates the transcription of pro-inflammatory genes in macrophages, which can be effectively suppressed by the RIPK1 inhibitor [20]. Therefore, it is necessary to find out the influence of RIPK1 signaling on neutrophils and endothelial cells, with a specific focus on the entangled interaction between the necroptotic pathway and inflammatory processes during ALI.

In this study, we investigated that RIPK1 inhibitor significantly alleviated lung inflammation and diminished neutrophil infiltration, thereby ameliorating histological lung damage. Additionally, we discovered that RIPK1 inhibitor could not only impede both neutrophil activation and endothelial cell damage but also disrupt the cycle of mutually reinforcing activation between them. More importantly, the protective effect of RIPK1 inhibition is not solely related to necroptosis. These findings offered evidence for a new mechanism of RIPK1 inhibitor on ALI treatment, suggesting a role in direct inhibition of neutrophil activation and endothelial cell damage through a cell-death-independent manner.

## Results

### RIPK1 inhibitor alleviates lung pathological damage in LPS-induced ALI mice

**Fig. 1:**
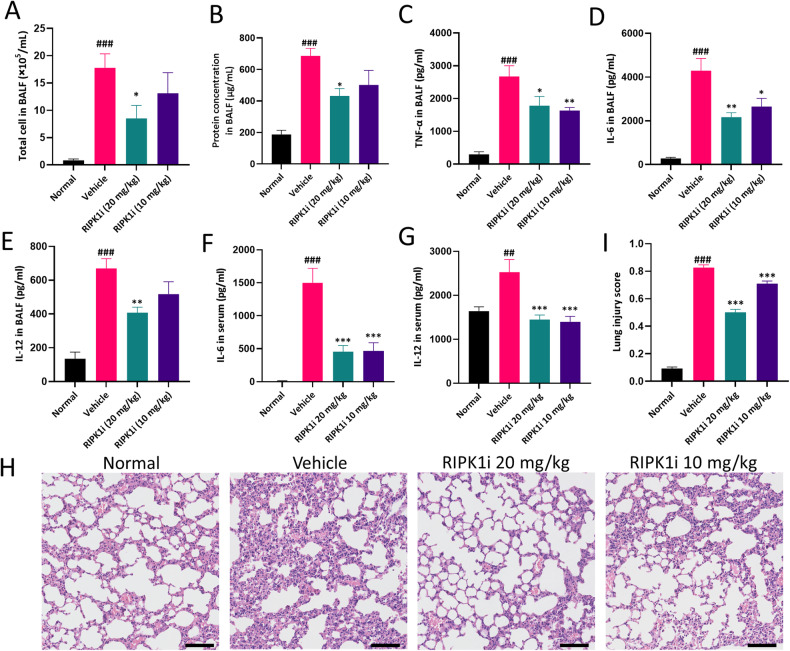
RIPK1 inhibitor alleviates lung pathological damage in LPS-induced ALI mice. **A**, **B** The total number of cells and protein content in BALF were determined. **C**–**E** The level of cytokines (TNF-α, IL-6 and IL-12) in BALF were measured by ELISA. **F**, **G** The level of cytokines (IL-6 and IL-12) in serum were measured by ELISA. **H** Representative images of the lung tissue sections stained with H&E were shown. Scale bars, 100 μm. **I** Statistical analysis of histological scores. *n* = 6 mice per group. All data are presented as means ± SEM. ^*^P < 0.05, ^**^P < 0.01, ^***^P < 0.001 compared to vehicle group. ^#^*P* < 0.05, ^##^*P* < 0.01, ^###^*P* < 0.001 compared to normal group.

To explore the role of RIPK1 in lung injury, we induced acute lung injury in mice through aerosol inhalation of LPS and orally intervened with RIPK1 inhibitor GSK2982772 (20 mg/kg, 10 mg/kg). According to existing literature, acute lung injury can lead to alveolar and microvascular structure damage, resulting in a significant influx of inflammatory cells into the BALF, and inflammatory mediators release from abnormally increased cells [1]. Additionally, the increased number of infiltrating cells and plasma protein leakage contributed to an elevated protein content within BALF. As local lung inflammation spreads systemically, there is a corresponding increase in the levels of inflammatory cytokines within the serum. In our study, we collected BALF and observed a pronounced increase in the cell count and the protein level in the vehicle group. Notably, the abnormal increase in cell population and protein level significantly decreased upon RIPK1 inhibition (Fig. 1A, B). We also utilized ELISA to measure the levels of inflammatory cytokines in BALF and serum. The vehicle group exhibited elevated levels of IL-6, IL-12, and TNF-α in BALF, but these levels decreased after the administration of the RIPK1 inhibitor (Fig. 1C–E). Parallel changes in the levels of cytokines IL-6 and IL-12 were observed in serum (Fig. 1F, G). To provide a more comprehensive assessment of lung tissue damage, we conducted histopathological observations on the mice. H&E staining of lung tissue sections revealed increased infiltration of immune cells, alveolar collapse, and localized pulmonary congestion in the vehicle group, all of which were alleviated by the administration of the RIPK1 inhibitor (Fig. 1H, I). Taken together, these findings suggest that inhibiting RIPK1 activity has the potential to ameliorate tissue damage and reduce the inflammatory response in mice with acute lung injury.

### RIPK1 inhibitor ameliorates neutrophil infiltration and reduces the expression of chemokines in the lung tissue of ALI mice

**Fig. 2:**
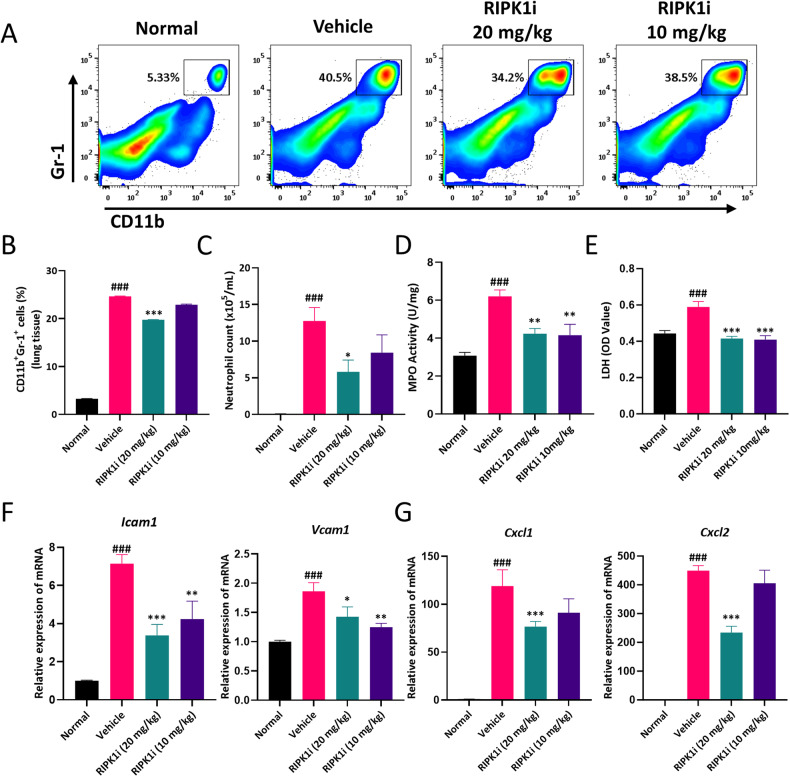
RIPK1 inhibitor ameliorates neutrophil infiltration and reduces the expression of chemokines in the lung tissue of ALI mice. **A**, **B** Flow cytometry analysis of CD11b^+^Gr-1^+^ neutrophils in the lung (gated on FVD^int^ viable single cells) and quantification analysis of neutrophils (% of total cell suspension). **C** The cell number of CD11b^+^Gr-1^+^ neutrophils in BALF by FACS. **D** MPO activity in the lung tissue was determined. **E** The production of LDH in serum was detected. Gene expressions of the adhesion molecules (**F**) and chemokines (**G**) were determined in lung tissue. *n* = 6 mice per group. All data are presented as means ± SEM. ^*^P < 0.05, ^**^P < 0.01, ^***^P < 0.001 compared to vehicle group. ^#^*P* < 0.05, ^##^*P* < 0.01, ^###^*P* < 0.001 compared to normal group.

During acute lung injury, chemotaxis attracts a significant number of immune cells to the lung tissue. Notably, neutrophils have a distinct advantage due to their rapid migration. We analyzed the proportion of neutrophils (CD11b^+^Gr-1^+^) and found that the number of neutrophils significantly increased in the LPS model group, but decreased after intervention with the RIPK1 inhibitor (Fig. 2A, B). Similar results were observed in BALF (Fig. 2C). Moreover, we assessed the levels of myeloperoxidase (MPO) in lung tissue, which is primarily expressed in neutrophils and serves as a biomarker for their presence. We observed an increase in MPO levels in the lung tissue homogenate of mice with acute lung injury, but these levels were reduced following treatment with the RIPK1 inhibitor (Fig. 2D). Lactate dehydrogenase (LDH) can serve as an indicator of tissue oxidative stress and cell death. Therefore, we measured LDH levels and found that the RIPK1 inhibitor could decrease LDH levels in the serum (Fig. 2E). The recruitment of immune cells from the peripheral immune system to lung tissue involves various adhesion molecules and chemokines. We utilized qPCR to assess the gene expression levels of the adhesion molecules ICAM-1 and VCAM-1. Our findings revealed that their expression was reduced in lung tissue following administration with the RIPK1 inhibitor (Fig. 2F). Furthermore, we examined the gene expression of various chemokines in lung tissue, including neutrophils attractor Cxcl1 and Cxcl2, which was substantially suppressed after RIPK1 inhibitor intervention (Fig. 2G). These results suggested that the RIPK1 inhibitor could reduce neutrophil infiltration and downregulate the expression of chemotactic and adhesion molecules, thereby alleviating the inflammatory response in lung tissue.

### RIPK1 inhibitor restrains ROS production and necroptosis in neutrophils

**Fig. 3:**
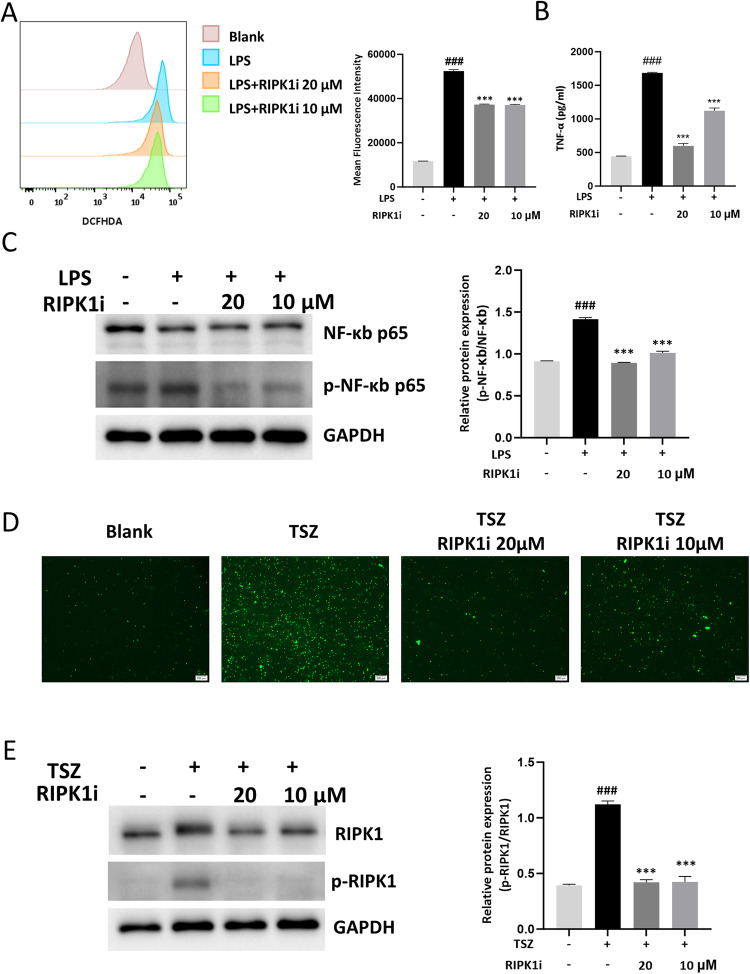
RIPK1 inhibitor restrains ROS production and necroptosis in neutrophils. Neutrophils were incubated with 1 μg/mL LPS or TSZ (100 ng/mL TNF-α, 50 nM SM-164, 20 μM Z-VAD-FMK) and the corresponding concentration of GSK2982772 (20 μM, 10 μM) for 3 h. **A** Representative FACS plots and statistical analysis were conducted to assess the ROS production in LPS-primed neutrophils. **B** The level of TNF-α in the culture of neutrophils was determined by ELISA. **C** Representative Western blot and statistical analysis of NF-κb p65 and p-NF-κb p65 in LPS-primed neutrophils. **D** Representative images of TSZ-primed neutrophils stained with Sytox Green. Scale bars, 100 μm. **E** Representative Western blot and statistical analysis of RIPK1 and p-RIPK1 in TSZ-primed neutrophils. These results are representative of three independent experiments. All data are presented as means ± SEM. ^*^P < 0.05, ^**^P < 0.01, ^***^P < 0.001 compared to LPS or TSZ group. ^#^*P* < 0.05, ^##^*P* < 0.01, ^###^*P* < 0.001 compared to blank group.

Based on the aforementioned in vivo research findings, we observed that the RIPK1 inhibitor exhibits a beneficial effect on acute lung injury. In response to the stimulation of pathogen-associated molecular patterns (PAMPs) present in the pulmonary microenvironment, neutrophils release intracellular reactive oxygen species (ROS) into the extracellular space, thereby exacerbating lung tissue damage [21]. To further investigate this phenomenon, we isolated mature neutrophils from bone marrow and subjected them to in vitro stimulation with LPS (1 μg/mL). We observed a significant increase in intracellular ROS levels and a substantial secretion of TNF-α in neutrophils upon LPS stimulation. However, the administration of RIPK1 inhibitor effectively suppressed these phenomena (Fig. 3A, B). Additionally, we labeled dead neutrophils using Sytox Green and found that LPS stimulation did not result in significant neutrophil death (Fig. S1). This suggests that the inhibitory effect of the RIPK1 inhibitor on neutrophil activation may not be related to necroptosis. ROS, which induce oxidative stress and inflammatory responses, have been reported to activate the NF-κB pathway. We conducted Western blot analysis to investigate the intracellular proteins in LPS-stimulated neutrophils and observed an increase in p-P65, indicating activation of the NF-κB pathway. However, upon intervention with the RIPK1 inhibitor, we observed a decrease in p-P65 (Fig. 3C). According to existing literature, RIPK1 plays a crucial role in necroptosis, resulting in the generation of a substantial amount of ROS. We stimulated neutrophils with TSZ (TNF-α + SM164 + Z-VAD) to induce necroptosis and observed rapid cellular demise, which was significantly attenuated by RIPK1 inhibitor (Fig. 3D). Moreover, the pronounced phosphorylation of RIPK1 indicated the occurrence of necroptosis, which was notably inhibited by treatment with the RIPK1 inhibitor (Fig. 3E). Furthermore, we examined the NF-κB pathway under TSZ stimulation and found that RIPK1 inhibitor could simultaneously reduce the expression of p-P65 (Fig. S2). The above findings demonstrated that the intervention of RIPK1 inhibitor could suppress the activation of neutrophils, which was manifested by the inhibition of the NF-κB pathway which is not solely dependent on necroptosis.

### RIPK1 inhibitor maintains the barrier function in TNF-α-primed vascular endothelial cells

**Fig. 4:**
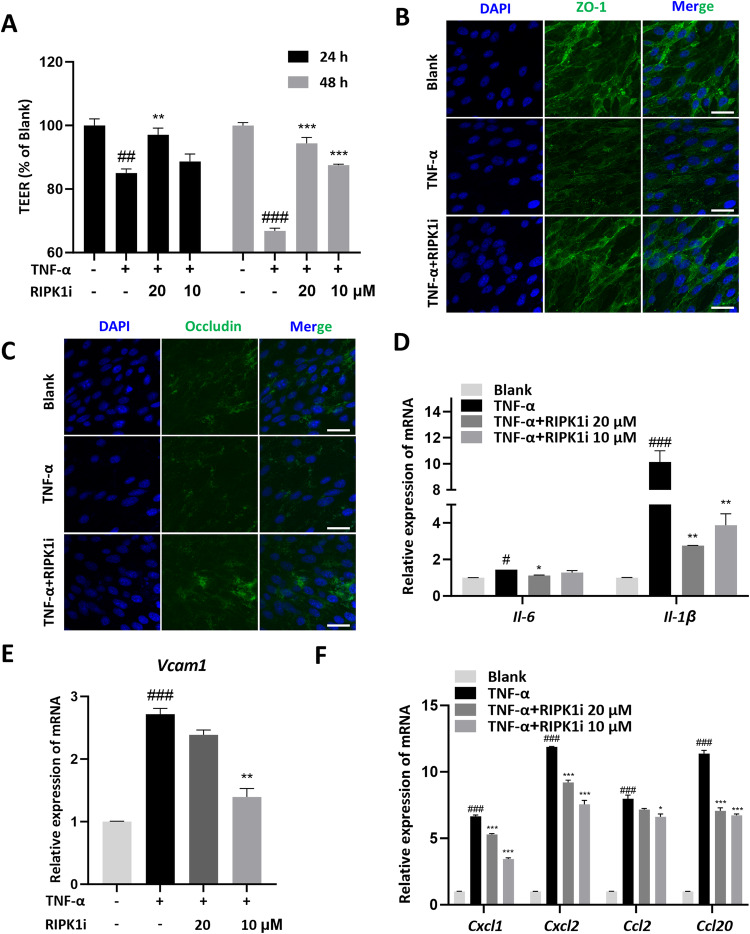
RIPK1 inhibitor maintains the barrier function in TNF-α-primed vascular endothelial cells. bEnd.3 cells were incubated with 100 ng/mL TNF-α and the corresponding concentration of GSK2982772 (20 μM, 10 μM) for 24/48 h. **A** Transepithelial electrical resistance (TEER) of TNF-α-primed bEnd.3 cells were determined. **B**, **C** Immunofluorescence analysis of tight junctions ZO-1 and Occludin in TNF-α-primed bEnd.3 cells for 24 h (scale bars, 20 μm). Gene expressions of cytokines (**D**), adhesion molecules (**E**), and chemokines (**F**) were determined in TNF-α-primed bEnd.3 cells for 24 h. These results are representative of three independent experiments. All data are presented as means ± SEM. ^*^P < 0.05, ^**^P < 0.01, ^***^P < 0.001 compared to TNF-α group. ^#^*P* < 0.05, ^##^*P* < 0.01, ^###^*P* < 0.001 compared to blank group.

Vascular endothelial cells play a key role in the progression of lung injury. The barrier damage of vascular endothelial cells leads to leakage of vascular contents and secretion of inflammatory cytokines, chemokines, and adhesion molecules, resulting in changes in vascular permeability and infiltration of immune cells into the alveolar space [22]. In the acute lung injury mouse model, LPS acts as a primary instigator of lung damage. On that account, we specifically targeted bEnd.3 cells, vascular endothelial cells, with LPS treatment. Contrary to our expectations, the gene expression levels of inflammatory cytokines, chemokines, and adhesion molecules in vascular endothelial cells appeared to be only slightly changed by exposure to LPS (Fig. S3). Moving forward, we replicated an intensely inflammatory environment within the lungs, stimulating the cells using TNF-α for evaluation. Notably, we observed that the diminished TEER values, indicative of endothelial barrier damage, were recovered upon administering the RIPK1 inhibitor (Fig. 4A). Further, we assessed the structural integrity of endothelial cells by examining ZO-1 and Occludin, popularly recognized markers of tight junctions. Our results intriguingly suggested that the tight junction disruption triggered by TNF-α could be mitigated by the RIPK1 inhibitor (Fig. 4B, C). Furthermore, when endothelial cells were stimulated solely with TNF-α, the RIPK1 inhibitor manifested a certain inhibitory impact on endothelial cell activation, evident by the decreased expression of inflammatory cytokines (IL-6 and IL-1β), adhesion molecules (VCAM-1), and chemotactic factors (CXCL1, CXCL2, CCL2, and CCL20) (Fig. 4D–F). Moreover, the effects of RIPK1 inhibitor were more likely to be dependent on the NF-κB pathway instead of the necroptosis pathway in this condition (Fig. S4). The inhibition of NF-κB activity is similar to that in neutrophils and inhibition of overall p-P65 levels could likewise be found in mouse lung tissue (Fig. S5). In conclusion, our findings underscore that the RIPK1 inhibitor could directly safeguard the barrier function of endothelial cells and curb their activation.

### RIPK1 inhibitor prevents vascular endothelial cell dysfunction induced by activated neutrophils

**Fig. 5:**
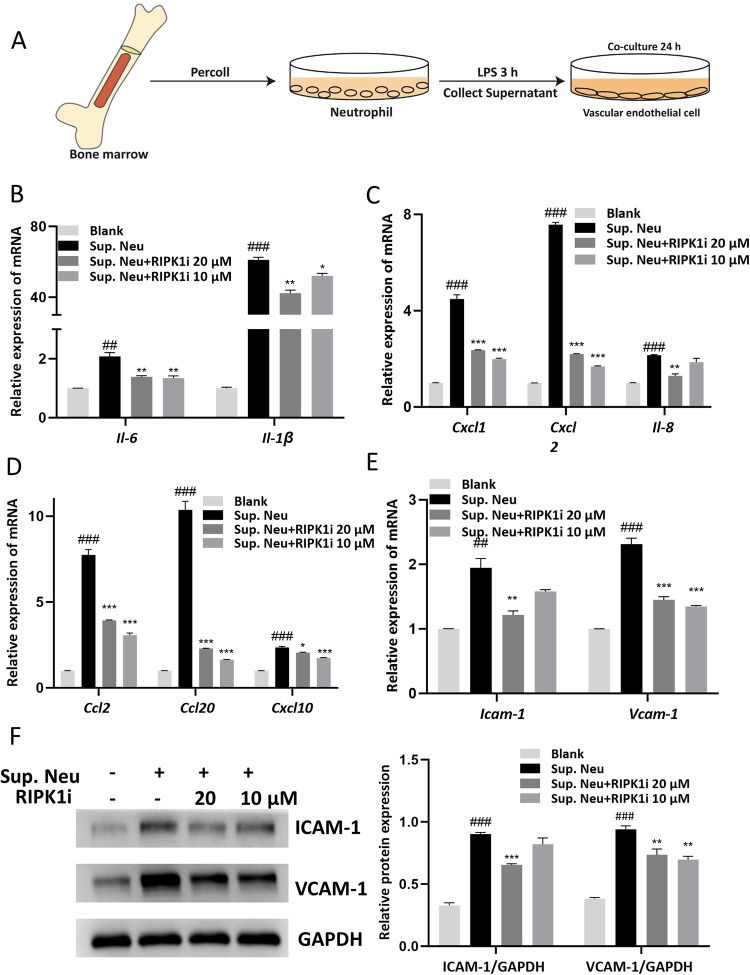
RIPK1 inhibitor prevents vascular endothelial cell dysfunction induced by activated neutrophils. **A** The flow diagram of neutrophil-vascular endothelial cell co-cultured system. Neutrophils were isolated from mouse bone marrow and incubated with 1 μg/mL LPS. After 3 h, the supernatant of neutrophils and GSK2982772 (20 μM, 10 μM) were added to the bEnd.3 cells for 24 h. Gene expressions of cytokines (**B**), chemokines (**C**, **D**) and adhesion molecules (**E**) were determined in co-cultured bEnd.3 cells. **F** Representative Western blot and statistical analysis of ICAM-1 and VCAM-1 in co-cultured bEnd.3 cells. These results are representative of three independent experiments. All data are presented as means ± SEM. ^*^P < 0.05, ^**^P < 0.01, ^***^P < 0.001 compared to Sup. Neu group. ^#^*P* < 0.05, ^##^*P* < 0.01, ^###^*P* < 0.001 compared to blank group.

Based on the above results, we found that RIPK1 inhibitor has a direct effect on neutrophils and vascular endothelial cells. To investigate the potential of RIPK1 inhibitor in modulating the interaction between neutrophils and vascular endothelial cells under pathological conditions, we co-cultured the supernatant of LPS-stimulated neutrophils with bEnd.3 cells and treated them with RIPK1 inhibitor (Fig. 5A). We observed that compared to the blank group, the supernatant from LPS-stimulated neutrophils upregulated the expression levels of pro-inflammatory factors IL-6 and IL-1β in bEnd.3 cells. However, RIPK1 inhibitor treatment abolished the stimulatory action (Fig. 5B). In addition, the culture supernatant of LPS-stimulated neutrophils upregulated the expression of chemokines such as IL-8, CXCL1, and CXCL2, known to be chemotactic factors for neutrophils (Fig. 5C). Moreover, an increase in CCL2, CCL20, and CXCL10 expression, chemotactic factors for monocytes, was observed (Fig. 5D). Remarkably, treatment with RIPK1 inhibitor resulted in a significant reduction in the expression of these chemokines. Considering the crucial role of immune cell migration towards target tissues, adhesion to vascular endothelial cells is vital. Through qPCR and Western Blot analysis, we observed that the culture supernatant from LPS-stimulated neutrophils upregulated the expression of vascular endothelial adhesion molecules ICAM-1 and VCAM-1. However, upon treatment with RIPK1 inhibitor, the expression of these adhesion molecules decreased both at the gene and protein levels (Fig. 5E, F). In addition, we co-cultured vascular endothelial cells with a neutrophil medium containing RIPK1 inhibitor and found that it could similarly inhibit the activation of endothelial cells (Fig. S6A, B). Based on the results obtained, we discovered that inhibiting RIPK1 effectively disrupts the positive feedback between neutrophils and vascular endothelial cells, thereby mitigating the detrimental effects on the pulmonary immune microenvironment.

### Long-term treatment with RIPK1 inhibitor attenuates innate immune cell infiltration and activation in LPS-induced lung injury

**Fig. 6:**
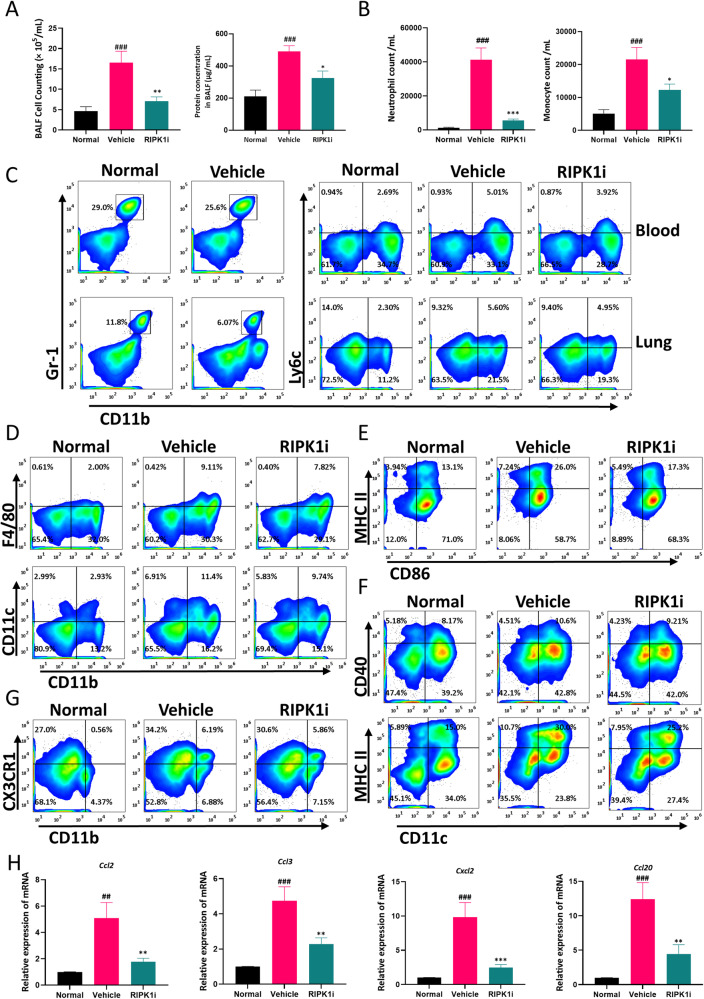
Long-term treatment with RIPK1 inhibitor attenuates innate immune cell infiltration and activation in LPS-induced lung injury. **A** The total number of cells and protein content in BALF were determined in the long-term treatment ALI model. **B** The cell number of CD11b^+^Gr-1^+^ neutrophils and CD11b^+^Ly6C^+^ monocytes in BALF by FACS. **C** Flow cytometry analysis of CD11b^+^Gr-1^+^ neutrophils and CD11b^+^Ly6c^+^ monocytes in the peripheral blood and lung (gated on FVD^int^ viable single cells). **D** Flow cytometry analysis of CD11b^+^F4/80^+^ macrophages and CD11b^+^CD11c^+^ dendritic cells in the lung (gated on FVD^int^SiglecF^-^ viable single cells). **E**, **F** Flow cytometry analysis of CD86^+^MHC II^+^ cells (gated on CD11b^+^F4/80^+^ macrophages), CD11c^+^CD40^+^ cells and CD11c^+^MHC II^+^ cells (gated on FVD^int^CD11b^+^ viable single cells) in lung. **G** Flow cytometry analysis of CD11b^+^CX3CR1^+^ cells in the lung (gated on FVD^int^ viable single cells). **H** Gene expressions of the chemokines were determined in the lung. *n* = 6 mice per group. All data are presented as means ± SEM. ^*^P < 0.05, ^**^P < 0.01, ^***^P < 0.001 compared to vehicle group. ^#^*P* < 0.05, ^##^*P* < 0.01, ^###^*P* < 0.001 compared to normal group.

The above data suggests a correlation between RIPK1 and the improvement of acute lung injury, as well as its intervention with neutrophils and vascular endothelial cells. To investigate the therapeutic effects of continuous treatment with RIPK1 inhibitor, we induced lung injury in mice by bronchial instillation of LPS (2 mg/kg) and administered daily RIPK1 inhibitor treatment. The results demonstrated that continuous administration of the RIPK1 inhibitor reduced the total cell count and protein concentration in the BALF (Fig. 6A). Furthermore, we performed cell counts on neutrophils and monocytes in the BALF and found that the RIPK1 inhibitor decreased the infiltration of these immune cells (Fig. 6B). In contrast to acute lung injury occurring in the short term, the overall proportion of neutrophils in lung tissue and peripheral blood was no longer significantly dominant. Conversely, monocytes showed a gradual increase and could be reduced by RIPK1 inhibitor in mice seven days after injury. (Fig. 6C). In addition, when examining the proportions of monocyte-derived cells in the lung tissue, we observed an increase in CD11b^+^F4/80^+^ macrophages and CD11b^+^CD11c^+^ dendritic cells in the injured lung tissue. However, RIPK1 inhibitor treatment resulted in a reduction in their expression levels (Fig. 6D). Moreover, we assessed the surface activation markers on macrophages and dendritic cells and noticed an increase in MHC II^+^CD86^+^ M1-like macrophages (gate on CD11b^+^F4/80^+^) and MHC II^+^CD40^+^ activated dendritic cells (Fig. 6E, F). Interestingly, RIPK1 inhibitor intervention led to a decrease in the proportion of activated immune cells. Additionally, our examination of the expression of the chemokine receptor CX3CR1 on CD11b^+^ mononuclear cells in lung tissue revealed an increased expression level in the model group but a reduced proportion upon RIPK1 inhibitor treatment (Fig. 6G). Finally, we analyzed the gene expression of chemokines in lung tissue and found elevated levels in the model group, which were significantly decreased by the RIPK1 inhibitor (Fig. 6H). These findings indicate that with the development of ALI, the dominant role of neutrophils is diminished, and monocyte-derived cells such as macrophages and dendritic cells become more prevalent and undergo activation. Moreover, the role of RIPK1 inhibitor in disrupting the immune microenvironment homeostasis by breaking the positive feedback loop between neutrophils and vascular endothelial cells further substantiates its effectiveness as a potential intervention for lung injury.

## Discussion

The lung is a vital organ that requires a delicate network of epithelial and endothelial cells to maintain their function. They allow for the efficient exchange of carbon dioxide with oxygen while being exposed to environmental stressors such as pathogens and aerosol toxins [23]. Consequently, severe damage to lung tissue caused by various triggers not only poses a threat to human survival but also significantly increases mortality rates [24]. ALI is characterized by the disruption of the alveolar-capillary barrier, which is caused by the death or dysfunction of alveolar epithelial cells and/or pulmonary microvascular endothelial cells, as well as the activation of neutrophils [1, 25, 26]. Over the past few decades, the crucial role of RIPK1 as a key target involved in necroptosis has been revealed in acute lung injury and disease, particularly in cells such as pulmonary epithelial cells and macrophages [16–19, 27–29]. Consistent with previous reports, we also discovered that RIPK1 inhibitor could protect lung epithelial cells from barrier damage induced by TNF-α, as evidenced by the maintenance of intercellular ZO-1 protein (Fig. S7). However, despite its expression in almost all cell types, the current research on RIPK1 in lung injury mainly focuses on necroptosis in pulmonary epithelial cells. Its specific role and mechanism in neutrophils and endothelial cells in ALI need further study. Here, we demonstrate that RIPK1 inhibitor could protect against LPS-induced ALI by suppressing neutrophil activation and mitigating damage to endothelial cells.

Neutrophils play a crucial pathological role in acute lung injury [26]. They sense damage in the alveolar microenvironment rapidly and migrate from the peripheral circulation to the alveolar space, where they hold a numerical advantage [30]. Once activated, neutrophils secrete significant quantities of ROS, neutrophil elastase, matrix metalloproteinases (MMPs), neutrophil extracellular traps (NETs) and cytokines [14, 21, 31]. ROS are crucial molecules in eliminating pathogens by oxidizing proteins, nucleic acids, and lipids [32]. However, once neutrophils are excessively activated or functionally impaired, ROS may surpass the cell’s clearance capacity, resulting in neutrophil death and the massive release of ROS into the extracellular environment, causing damage to lung tissues [33]. In addition, it has been reported that ROS could function as both second messengers of TNFα-induced cell death and modulators of signaling pathways [34]. The generation of ROS triggers necroptosis and plays a role in enhancing the formation of necrosome within the positive feedback loop [35, 36]. In the in vitro study, RIPK1 inhibitor administration significantly suppressed LPS-induced ROS and TNF-α generation, and blocked NF-κB pathway (Fig. 3A–C), without priming cell death in neutrophil (Fig. S1). Then we utilized the classical TSZ-induced necroptosis system and observed a quick response to necroptosis signal in neutrophils. Not surprisingly, inhibiting RIPK1 activity significantly reduced this form of cell death (Fig. 3D, E). ROS and RIPK1 are both closely associated with the NF-κB pathway, which is intricately linked to inflammatory responses and oxidative stress, as reported in the existing articles [37, 38]. Thus, RIPK1 inhibitor plays a protective role in neutrophils through traditional necroptosis signaling, while it is more importantly associated with direct inhibition of the NF-κB pathway and independent of necroptosis (Fig. 3C, Figs. S1−2).

The damage to endothelial cells is also a hallmark event in ALI [39]. In ALI, inflammatory mediators, ROS, and DAMPs released by inflammatory cells and alveolar epithelial cells can lead to endothelial cell injury and activation [22]. Damage to endothelial cells results in disruption of the alveolar-capillary barrier, and the extravasation of fluid and proteins into the interstitium and alveolar space, leading to pulmonary edema and pneumonia [13]. Meanwhile, activated endothelial cells also express adhesion molecules and chemotactic factors, attracting immune cells to adhere and migrate to the vessel wall, further fueling the inflammatory response [15]. Therefore, in this study, we selected the widely used endothelial cell line bEnd.3 for our in vitro experiments to simulate endothelial cell injury and activation stimulated with LPS or TNF-α [40–42]. Stimulation with TNF-α resulted in significant disruption of intercellular tight junctions and upregulation of inflammatory factors, adhesion molecules, and chemotactic factors (Fig. 4). The suppression of RIPK1 could inhibit the aforementioned changes, demonstrating a similar mechanism in neutrophils (Figs. S4–5). This indicates that RIPK1 inhibitor still plays an anti-inflammatory and protective role under death-unrelated stimuli. We believe that in the non-cell death state, RIPK1 inhibitor primarily acts through the inhibition of the NF-κB pathway, but further research is needed to explore the involvement of underlying molecular mechanisms. Furthermore, this study did not directly investigate the functional changes of endothelial cells under conditions of cell death. According to the hints of existing literature, some studies have reported that endothelial programmed necroptosis plays an important role in lung injury and brain injury, which warrants further investigation in future studies [43, 44]. Since LPS stimulation did not lead to noticeable activation or damage in endothelial cells (Fig. S3), we remain focused on exploring the interaction between neutrophils and endothelial cells. To simulate the immune microenvironment of ALI, we co-cultured endothelial cells with the culture supernatant of LPS-stimulated neutrophils. Interestingly, we found that the supernatant of LPS-stimulated neutrophils strongly induced the upregulation of inflammatory factors, chemotactic factors, and adhesion molecules in endothelial cells. Encouragingly, when we intervened with RIPK1 inhibitor, both before and after neutrophil stimulation, effective suppression of endothelial cell activation was clearly observed (Figs. 5, S6). The above in vitro findings strongly support the pharmacological effect and indicate the biological mechanisms of RIPK1 inhibitor in ALI model (Figs. 1, 2).

**Fig. 7:**
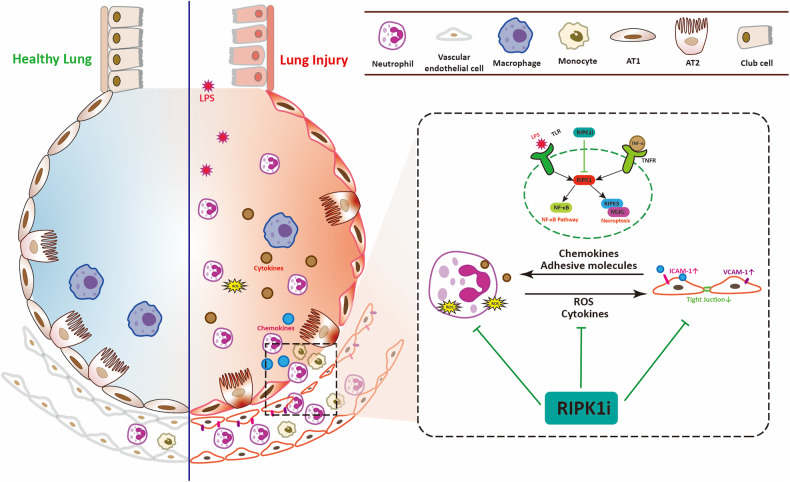
The schematic diagram of targeting RIPK1 ameliorates pulmonary injury by modulating the function of neutrophils and vascular endothelial cells.

In the clinical setting, ALI progresses to a syndrome in most patients within 72 h and poses a risk to nearly all patients within one week, particularly in those with pneumonia and sepsis [3]. To verify its long-term effect, RIPK1 inhibitor was administered to mice with pulmonary injury for one week. We found that systemic inflammation gradually subsided in mice, while the lungs remained in an inflammatory state locally (Fig. 6). Furthermore, as neutrophils decreased, monocytes began to play a dominant role in lung tissue [45]. As a result of monocyte differentiation in lung tissue, a large number of macrophages and dendritic cells infiltrated were activated in the lung tissue. Continuous treatment with RIPK1 inhibitor could suppress inflammation in the lungs and inhibit the infiltration and activation of monocyte-derived cells (Fig. 6), albeit not significantly. This observation suggests that the ameliorative effects of the RIPK1 inhibitor on lung injury may not be from its direct impact on these cell types. We think that RIPK1 inhibitor reshapes the pulmonary immune microenvironment, thereby influencing monocytes and monocyte-derived cells. Thus the beneficial effect of RIP1 inhibitor was promised both in the early and late stage in ALI (Fig. 7).

In summary, we have shown that RIPK1 inhibitor directly affects neutrophils and endothelial cells, disrupting the dysregulated pulmonary immune microenvironment caused by their “positive feedback” in ALI. Mechanistically, RIPK1 may exert its role in alleviating lung injury not only by inhibiting the necroptosis pathway but more importantly by suppressing the NF-κB pathway of target cell populations.

## Materials and methods

### Animals

C57BL/6J mice (6–8 weeks) were obtained from Shanghai Lingchang Biotechnology Co.Ltd. They were then housed and maintained in the specific pathogen-free (SPF) animal facilities of Shanghai Institute of Materia Medica. All experiments conducted in this study followed the guidelines set by the Association Assessment and Accreditation of Laboratory Animals Care International. All the procedures were carried out strictly following the animal care and use protocol (2022-01-TW-86) approved by the Institutional Animal Care and Use Committee (IACUC) at Shanghai Institute of Materia Medica.

### LPS-induced lung injury model

LPS-induced acute lung injury model in mice via nebulization [46, 47]: Mice were placed in the chamber of a nebulizer and exposed to 2.5 mg/mL LPS (L2880, Sigma-Aldrich, MO, USA) at a uniform rate. Nebulization was performed for 30 min with a 2 h pause in between. The second nebulization lasted for 30 min, followed by a 4 h waiting period before conducting endpoint procedures on the animals. GSK2982772 (MedChemExpress, New Jersey, USA) was prepared using 0.2% HPMC (Sigma-Aldrich) as a solvent, which was sonicated to form a homogeneous suspension. Prior to modeling, GSK2982772 was administered via oral gavage to the mice 30 min before. Mice were randomly divided into four groups (normal, vehicle receiving only LPS, and treatment receiving LPS + GSK2982772 (20 mg/kg, 10 mg/kg)) with six mice per group.

Bronchial instillation of LPS-induced lung injury therapeutic model [40, 48]: Mice were anesthetized and then administered 2 mg/kg of LPS via bronchial instillation. GSK2982772 administration was performed through oral gavage daily for 7 days. Mice were randomly divided into three groups (normal, vehicle receiving only LPS, and treatment receiving LPS + GSK2982772 (10 mg/kg)) with six mice per group.

### Lung histopathological examination

Mouse lung tissues were fixed in 4% paraformaldehyde, embedded in paraffin, and sectioned. To conduct histopathological analysis, the paraffin sections were stained with hematoxylin and eosin (H&E). The severity of histological lung injury was evaluated using the lung injury scoring system established by the American Thoracic Society in 2011 [49].

### Bronchoalveolar lavage fluid collection

After euthanizing the mice, the trachea was exposed and an 18 G sterile needle was inserted to facilitate the procedure. Subsequently, the bronchoalveolar lavage fluid (BALF) was collected by gently flushing the lungs with 0.8 mL of cold PBS for three cycles. To separate the supernatant from the cells, the collected BALF was then centrifuged at 1500 rpm for 5 min.

### Single-cell preparation and flow cytometry analysis

The lung tissues were placed in RPMI 1640 medium containing 10% PBS and prepared with a solution of 1.5 mg/mL type IV collagenase (Sigma-Aldrich) and 0.04 mg/mL DNase I (Roche, Mannheim, Germany). The mixture was then incubated at 37 °C for 1 h. The lung tissues were homogenized to obtain a suspension, and red blood cells were lysed using a lysis buffer (Beyotime Biotechnology, Shanghai, China). Subsequently, the suspension was sequentially passed through 100 μm, 70 μm, and 40 μm mesh filters to obtain a single-cell suspension of lung tissues.

For flow cytometric analysis, the single cells were first washed with PBS. To determine the viability of the cells, they were then incubated with a fixable viability dye, eFluor™ 780 (eBioscience, San Diego, CA, USA), at 4 °C for 30 min. To label the surface markers, the cells were blocked with an anti-CD16/CD32 monoclonal antibody (Thermo Fisher Scientific, MA, USA) to prevent non-specific binding. Subsequently, the cells were incubated at 4 °C for 30 min with appropriate antibody-fluorophore conjugates for staining. Flow cytometric analysis was carried out using the BD LSRFortessa. The acquired data were then analyzed using FlowJo V10 software (Treestar, Ashland, OR, USA).

### Isolation and treatment of bone marrow neutrophils

According to the literature [50], bone marrow was obtained from the tibias and femurs of C57BL/6 mice, and single-cell suspensions were prepared. Separately, Percoll (Cytiva, Washington DC, USA) gradients with densities of 52%, 64%, and 72% were prepared. The cell suspension was layered on top of the gradient and centrifuged at 1545 × *g* for 30 min. Neutrophils were collected from the interface between the 72 and 64% layers. Subsequently, neutrophils were incubated with 1 μg/mL LPS or TSZ (100 ng/mL TNF-α (Peprotech, London, UK), 50 nM SM-164 (MedChemExpress), 20 μM Z-VAD-FMK (MedChemExpress)) and the corresponding concentration of GSK2982772 (20 μM, 10 μM) for 3 h.

### Cell culture and treatment

The mouse cerebral microvascular endothelial cell line bEnd.3 and mouse lung epithelial cell line MLE-12 were purchased from the American Type Culture Collection (ATCC, Manassas, VA, USA). Cells were cultured in DMEM medium or DMEM/F12 (Gibco, Grand Island, NY, USA), containing 10% fetal bovine serum (HyClone, Logan, UT, USA), 100 μ/mL penicillin, and 100 µg/mL streptomycin. Cells were maintained in a humidified incubator with 5% CO_2_ at 37 °C. For cell treatment, bEnd.3 cells and MLE-12 cells were cultured with 1 μg/mL LPS or 100 ng/mL TNF-α, for 24 h.

### Enzyme-linked immunosorbent assay (ELISA)

Cytokines in BALF, serum, and cell culture supernatants were quantified by mouse TNF-α, IL-6 and IL-12p40 enzyme-linked immunosorbent assay (ELISA) kits (BD Pharmingen, San Diego, CA, USA).

### RNA isolation and real-time quantitative PCR

Total RNA was extracted from tissues and cells using the Total RNA Extraction Purification Kit (Tiangen, Beijing, China). The experimental procedure was strictly followed according to the instructions provided with the kit. Subsequently, cDNA was synthesized from the RNA samples using the cDNA Synthesis SuperMix Reverse Transcription Kit (Yeasen, Shanghai, China). Real-time quantitative PCR analysis was performed using SYBR Green Realtime PCR Master Mix (Yeasen) and the 7500 Fast Real-Time PCR System (Applied Biosystems, Foster City, CA, USA) with gene-specific primers. The sequences of primers for RT-qPCR are as Table S1.

### Western blot analysis

For tissue samples, the lung was homogenized in a lysis buffer containing sodium dodecyl sulfate (SDS, Beyotime) with protease and phosphatase inhibitors (Beyotime). As for cell samples, the cells were lysed by repeated pipetting using SDS lysis buffer. The protein samples were boiled for 10 min, electrophoresed in SDS-PAGE gel, and then transferred to nitrocellulose membranes (Bio-Rad, Hercules, CA, USA). The membranes were blocked with SuperBlock™ T20 blocking buffer (Thermo Fisher Scientific) and incubated with appropriately diluted primary antibodies. The signals were visualized using the ChemiDoc™ MP Imaging System (Bio-Rad).

### Immunofluorescent staining

bEnd.3 cells on coverslips were fixed in the fixing solution (Beyotime, China) for 15 min. After blocking buffer (Beyotime) treatment, cells were incubated with rabbit anti-ZO-1(Proteintech, Wuhan, China) and Occludin (Thermo Fisher Scientific) respectively overnight. The FITC conjugated anti-rabbit was added after washing with 1% PBS-Tween. After being incubated for 1 h at room temperature, the cells were washed and counterstained with DAPI. All images were captured by Leica TCS SPS CFSMP microscope (Wetzlar, Germany).

### Reactive oxygen species assay

Cells were collected and rinsed in PBS. Fresh FBS-free medium with H2DCFDA (Beyotime) was added and incubated at 37 °C for 20 min. Then the cells were detached in PBS, washed twice, and resuspended in PBS. ROS production was detected by flow cytometric analysis.

### Detection of dead cell

The cells were transferred to a fresh medium and incubated with 1 μM Sytox Green (Thermo Fisher Scientific) for 5 min. This fluorescent dye specifically stains dead cells. After the incubation period, the cells were immediately observed under a fluorescence microscope (Olympus IX73, Tokyo, Japan). The dead cells were identified by their green fluorescence signal.

### Myeloperoxidase and LDH measurement

The lung tissue was homogenized, and the myeloperoxidase (MPO) activity was measured using the Odianisidine method, as previously reported [51]. The results were expressed as activity units per milligram of protein.

The relative lactate dehydrogenase (LDH) content in the serum was measured following the instructions provided with the LDH assay kit (Beyotime). The optical density (OD) values were recorded.

### Transepithelial electrical resistance (TEER)

To measure the barrier function of endothelial cells, we seeded bEnd.3 cells on the apical surface of the transwell polyester membrane filter with a pore size of 0.4 μm. The integrity of the cell monolayers was determined using an epithelial Volt-Ohm Meter (Millicell ERS2).

### Statistical analysis

The data in this study were presented as Mean ± SEM. Statistical analysis of the data was performed using GraphPad Prism 9.0 software (San Diego, CA, USA), including one-way ANOVA followed by Dunnett’s multiple comparison test to determine the significance of differences between groups. A *P* value less than 0.05 was considered statistically significant.

### Supplementary information

Supporting Information

## Data Availability

The data analyzed during this study are included in this article and the supplemental data files.
