# Supplementary material for: RIPK1 inhibitor ameliorates pulmonary injury by modulating the function of neutrophils and vascular endothelial cells
Source: Cell Death Discov. 2024 Mar 23;10:152. doi: 10.1038/s41420-024-01921-8 (PMC10960796; doi:10.1038/s41420-024-01921-8)

**Supporting Information**

**RIPK1 inhibitor ameliorates pulmonary injury by modulating the function of neutrophils and vascular endothelial cells**

Tao Yang^1,2^, Cai-gui Xiang^1,2^, Xiao-han Wang^1,2^, Qing-qing Li^1^, Shu-yue Lei^1,2^, Kai-rong Zhang^4^, Jing Ren^3^, Hui-min Lu^1,2^, Chun-lan Feng^1^ and Wei Tang^1,2*^

^1^State Key Laboratory of Chemical Biology, Shanghai Institute of Materia Medica, Chinese Academy of Sciences, Shanghai 201203, China

^2^School of Pharmacy, University of Chinese Academy of Sciences, Beijing 100049, China

^3^School of Chinese Materia Medica, Nanjing University of Chinese Medicine, Nanjing 210000, China

^4^School of Pharmaceutical Science, Nanchang University, Nanchang 330006, China

**Supplementary Figure 1 to 7**

**Supplementary Table 1**

**Original Western Blot**


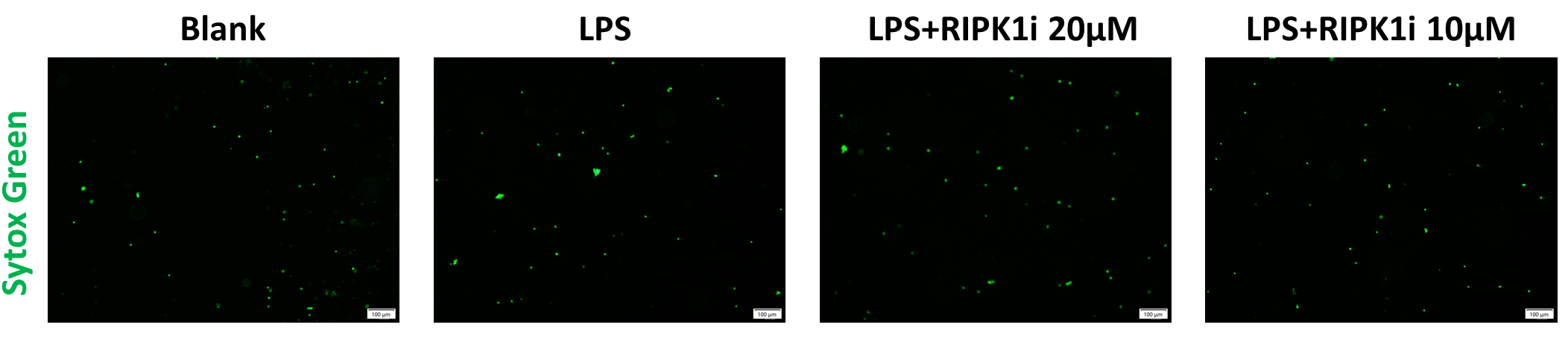
**Supplementary Fig. 1 Stimulation of neutrophils with LPS for 3 h does not result in significant cell death.** Representative images of LPS-primed neutrophils stained with Sytox Green. Scale bars, 100 μm.


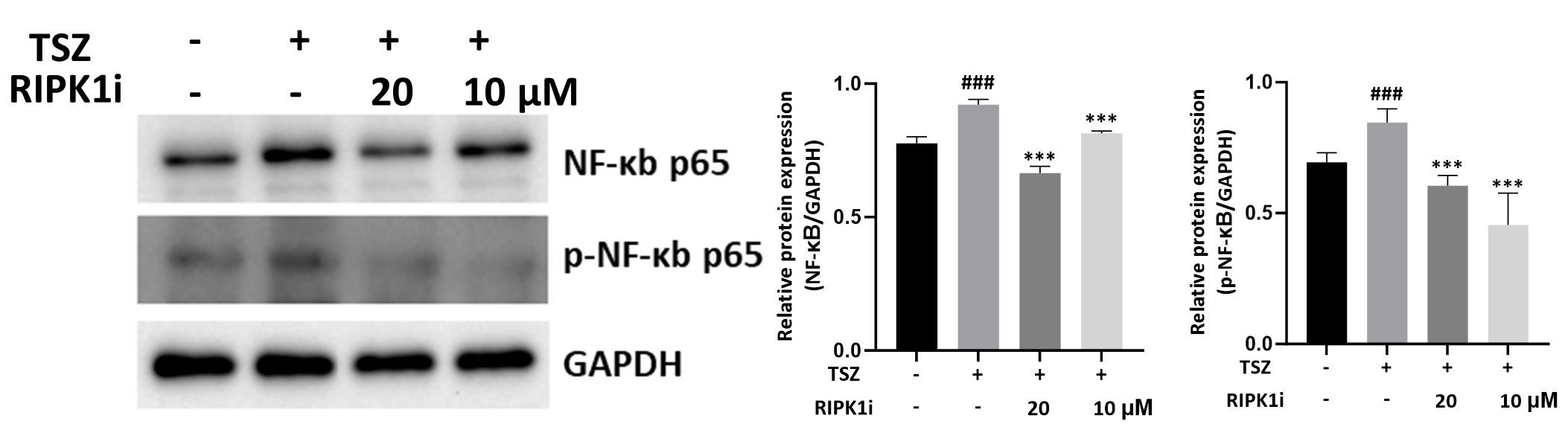
**Supplementary Fig. 2 RIPK1 inhibitor reduces the expression of both P65 and p-P65 in TSZ-primed neutrophils.** Neutrophils were incubated with TSZ (100 ng/mL TNF-α, 50 nM SM-164, 20 μM Z-VAD-FMK) and the corresponding concentration of GSK2982772 (20 μM, 10 μM) for 3 hours. Representative Western blot and statistical analysis of NF-κb p65 and p-NF-κb p65 in TSZ-primed neutrophils. All data are presented as means ± SEM. ^*^P < 0.05, ^**^P < 0.01, ^***^P < 0.001 compared to TSZ group. ^#^P < 0.05, ^##^P < 0.01, ^###^P < 0.001 compared to blank group.


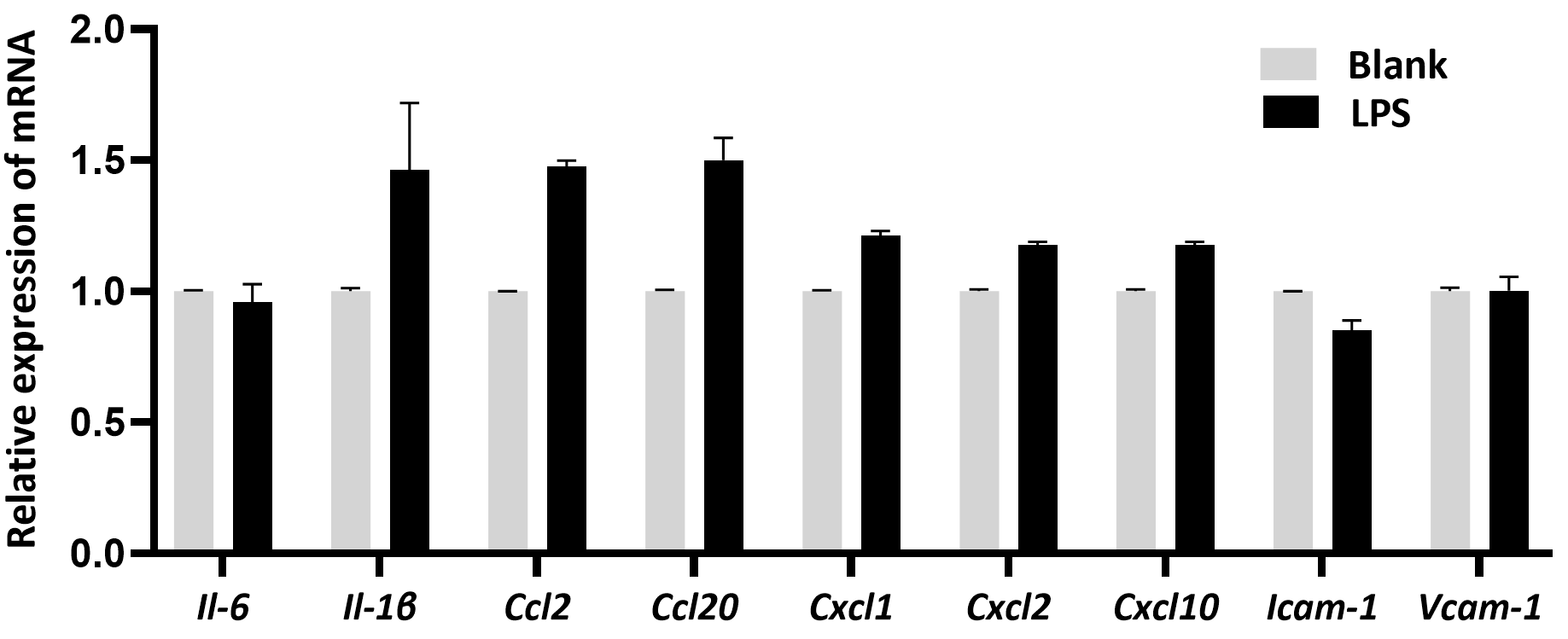
**Supplementary Fig. 3 Vascular endothelial cell function appeared to be only slightly increased or unchanged by exposure to LPS compared to TNF-α.** bEnd.3 cells were incubated with 1 μg/mL LPS for 24 hours. Gene expressions of cytokines, chemokines and adhesion molecules were determined in LPS-primed bEnd.3 cells.


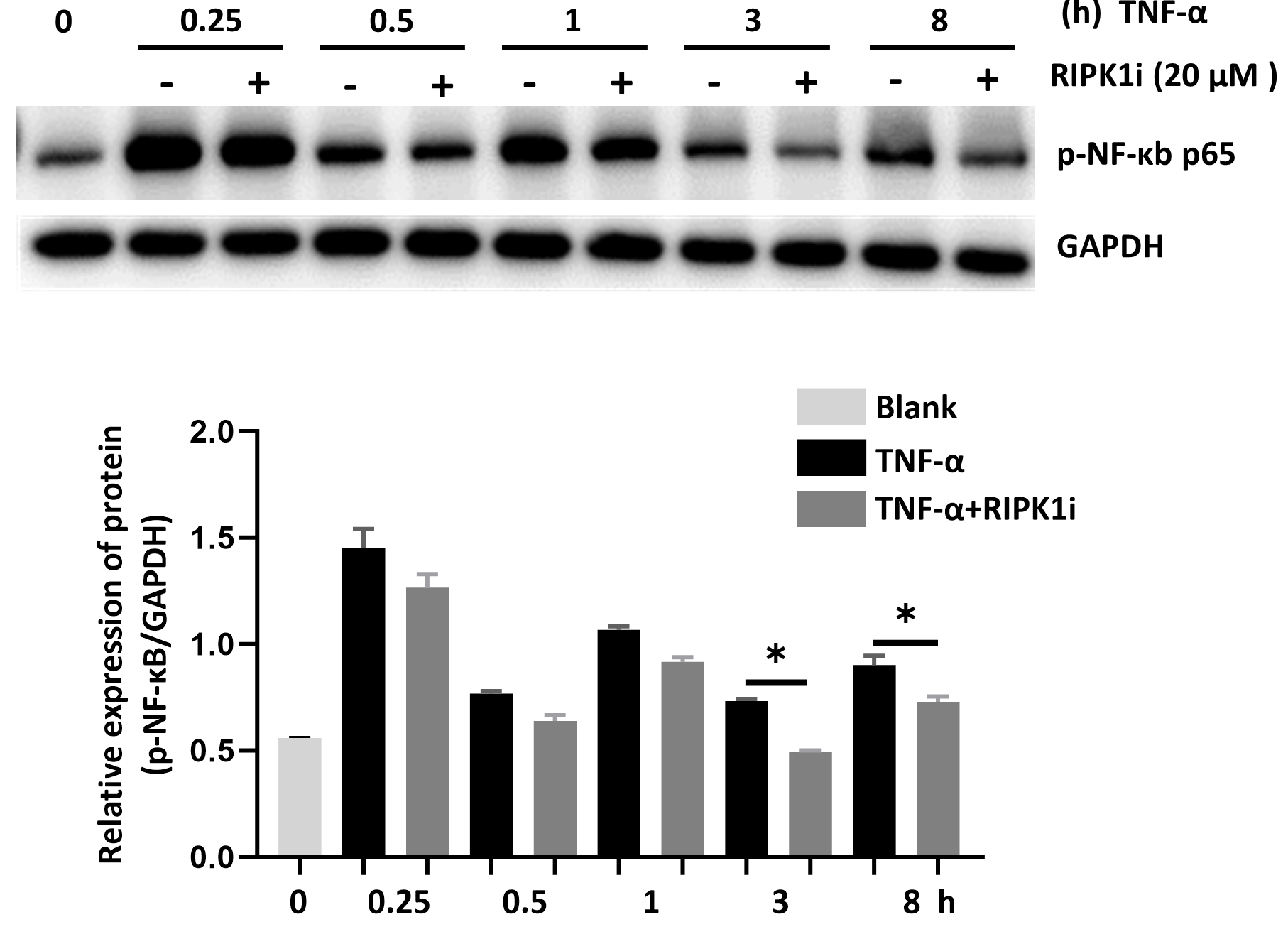
**Supplementary Fig. 4 RIPK1 inhibitor reduces the expression of p-P65 in TNF-α-primed** **bEnd.3 cells.** bEnd.3 cells were incubated with 100 ng/mL TNF-α and 20 μM GSK2982772 for the indicated time. Representative Western blot and statistical analysis of p-NF-κb p65 in TNF-α-primed bEnd.3 cells. All data are presented as means ± SEM. ^*^P < 0.05, compared to TNF-α group.


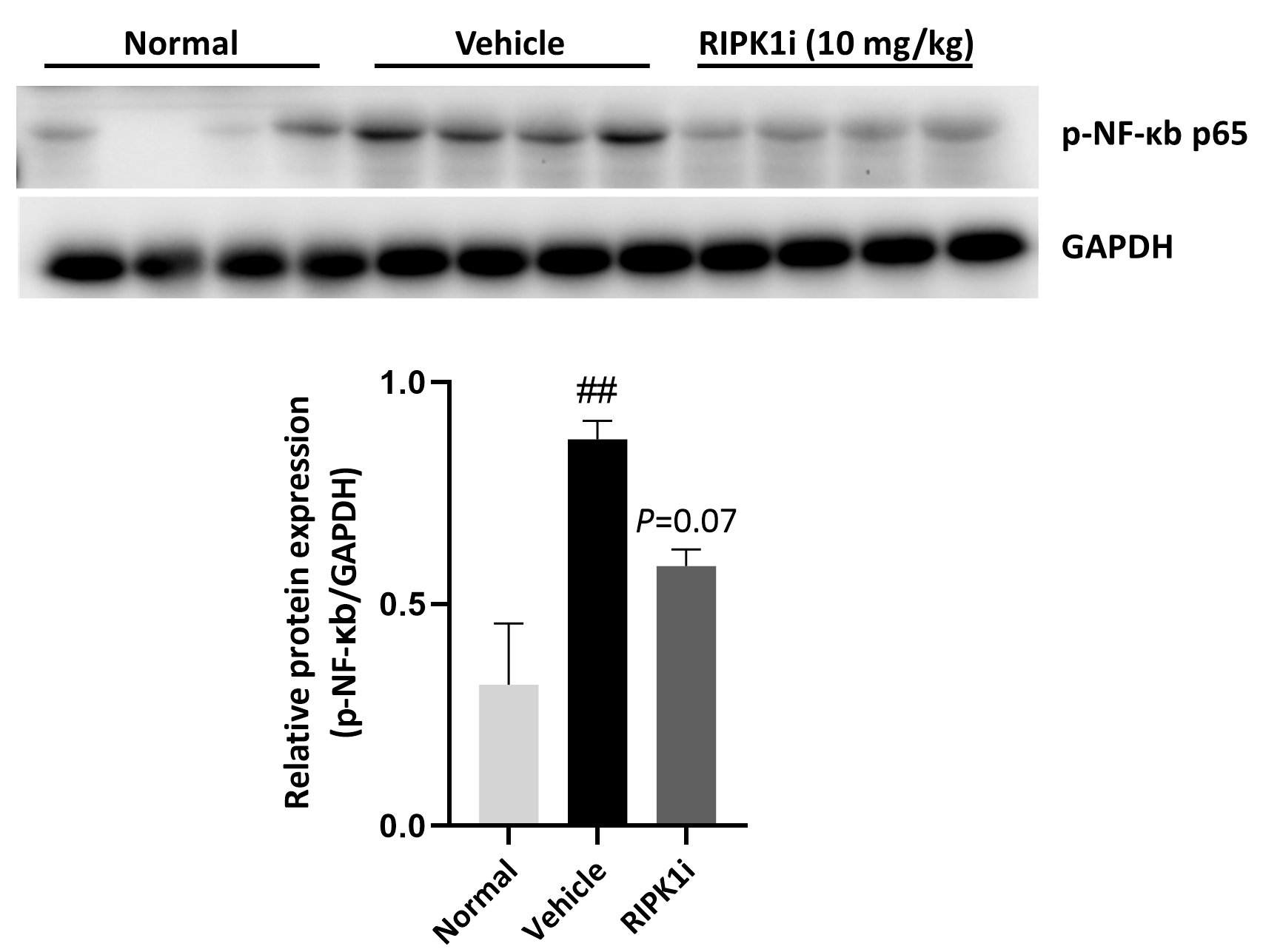
**Supplementary Fig. 5 RIPK1 inhibitor reduces the expression of p-P65 in ALI lung tissue.** Representative Western blot and statistical analysis of p-NF-κb p65. ^##^P < 0.01 compared to normal group.


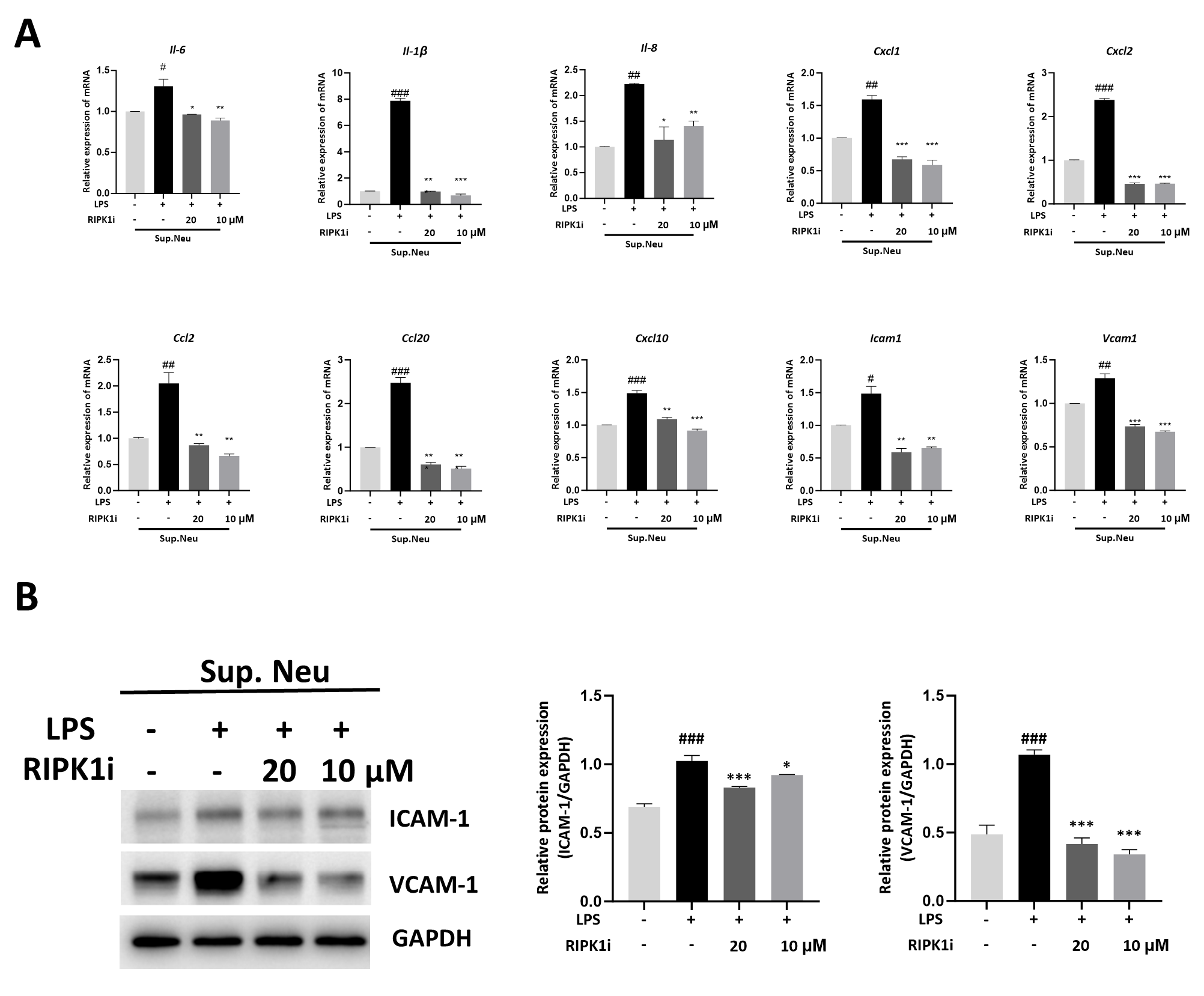
**Supplementary Fig. 6 The supernatant of neutrophil cultures pretreated with RIPK1 inhibitor could inhibit the activation of endothelial cells.** Neutrophils were isolated from mouse bone marrow and incubated with 1 μg/mL LPS and compounds. After 3 hours, the supernatant was centrifuged and added to the bEnd.3 cells for 24 hours. (A) Gene expressions of cytokines, chemokines and adhesion molecules were determined in co-cultured bEnd.3 cells. (B) Representative Western blot and statistical analysis of ICAM-1 and VCAM-1 in co-cultured bEnd.3 cells.


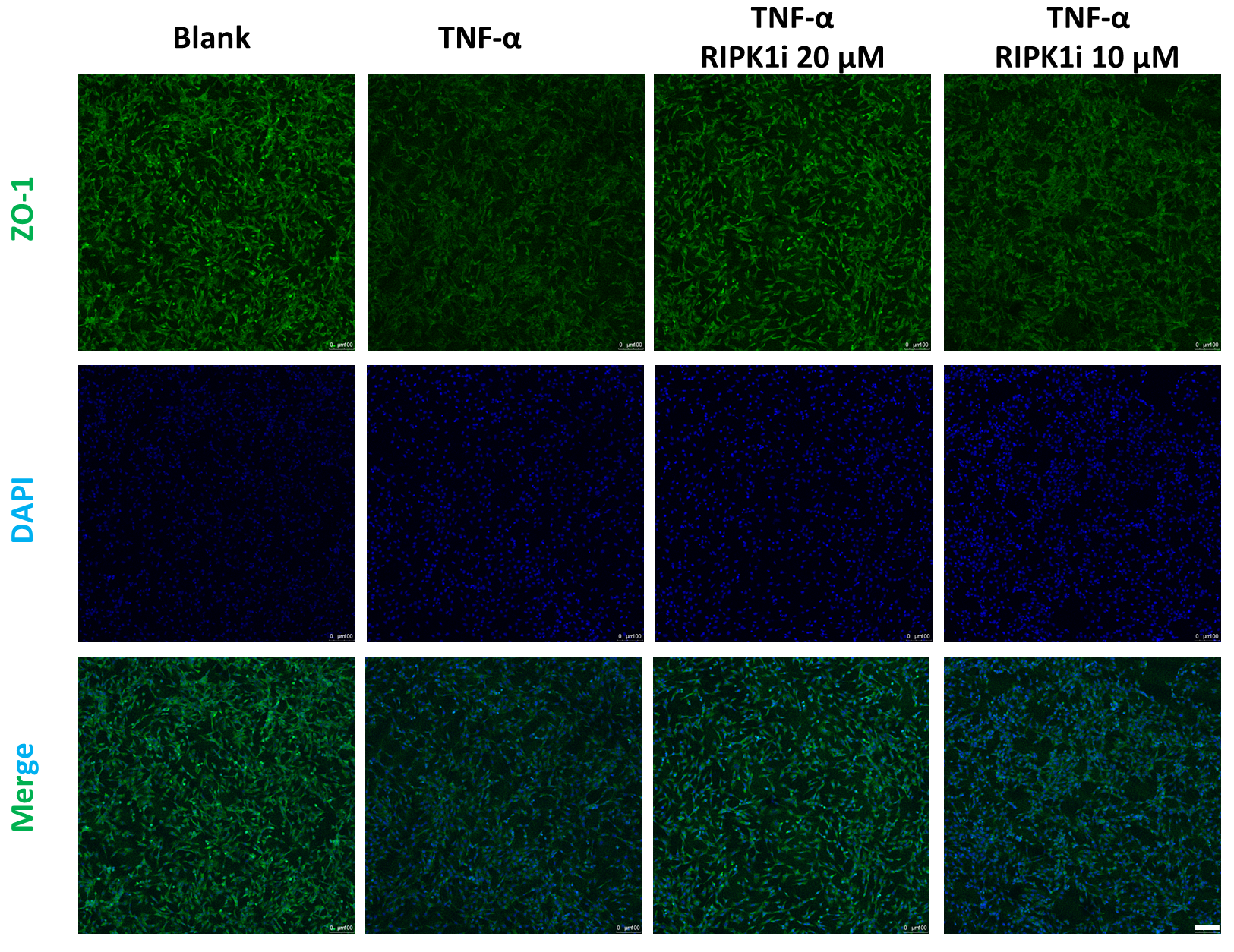
**Supplementary Fig. 7 RIPK1 inhibitor could protect lung epithelial cells from barrier damage induced by TNF-α.** MLE-12 cells were incubated with 100 ng/mL TNF-α and GSK2982772 (20 μM, 10 μM) for 24 h. Immunofluorescence analysis of tight junctions ZO-1 in TNF-α-primed MLE-12 cells (scale bars, 100 μm).

**Supplementary Table 1** Sequences of primers for real-time quantitative PCR.

| Gene | Sequence 5′–3′ |  |
| --- | --- | --- |
| Mouse | Forward | Reverse |
| *β*-Actin | GTGACGTTGACATCCGTAAAGA | GCCGGACTCATCGTACTCC |
| *Il-1β* | GAAATGCCACCTTTTGACAGTG | TGGATGCTCTCATCAGGACAG |
| *Il-6* | CTGCAAGAGACTTCCATCCAG | AGTGGTATAGACAGGTCTGTTGG |
| *Il-8* | TGTTGAGCATGAAAAGCCTCTAT | AGGTCTCCCGAATTGGAAAGG |
| *Icam-1* | GTGATGCTCAGGTATCCATCCA | CACAGTTCTCAAAGCACAGCG |
| *Vcam-1* | TTGGGAGCCTCAACGGTACT | GCAATCGTTTTGTATTCAGGGGA |
| *Ccl20* | ACTGTTGCCTCTCGTACATACA | GAGGAGGTTCACAGCCCTTTT |
| *Cxcl1* | ACTGCACCCAAACCGAAGTC | TGGGGACACCTTTTAGCATCTT |
| *Cxcl10* | CCAAGTGCTGCCGTCATTTTC | GGCTCGCAGGGATGATTTCAA |
| *Ccl2* | TAAAAACCTGGATCGGAACCAAA | GCATTAGCTTCAGATTTACGGGT |
| *Cxcl2* | TTCTCTGTACCATGACACTCTGC | CGTGGAATCTTCCGGCTGTAG |
| *Ccl3* | TGTACCATGACACTCTGCAAC | CAACGATGAATTGGCGTGGAA |


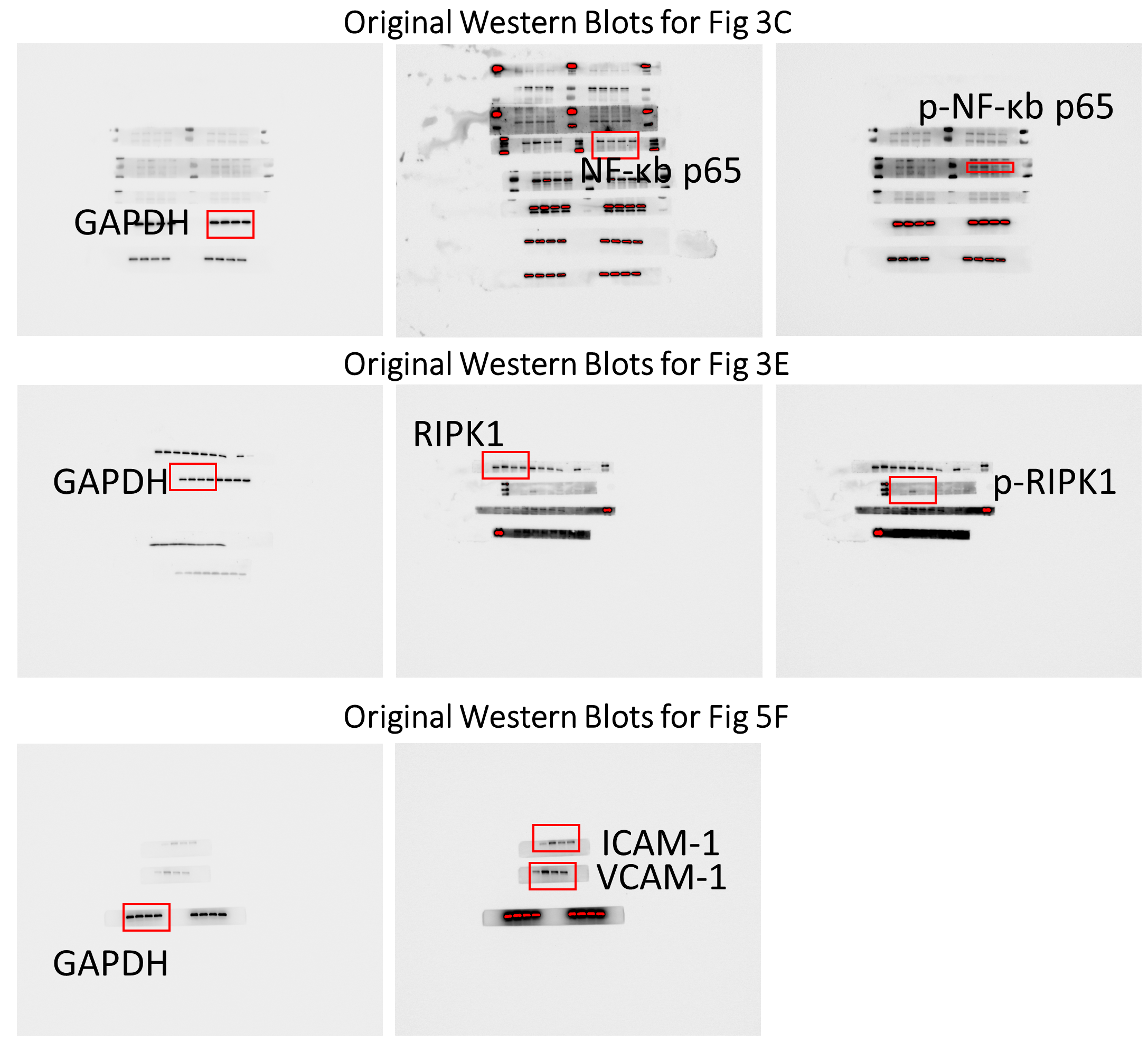
**Original Western Blot**


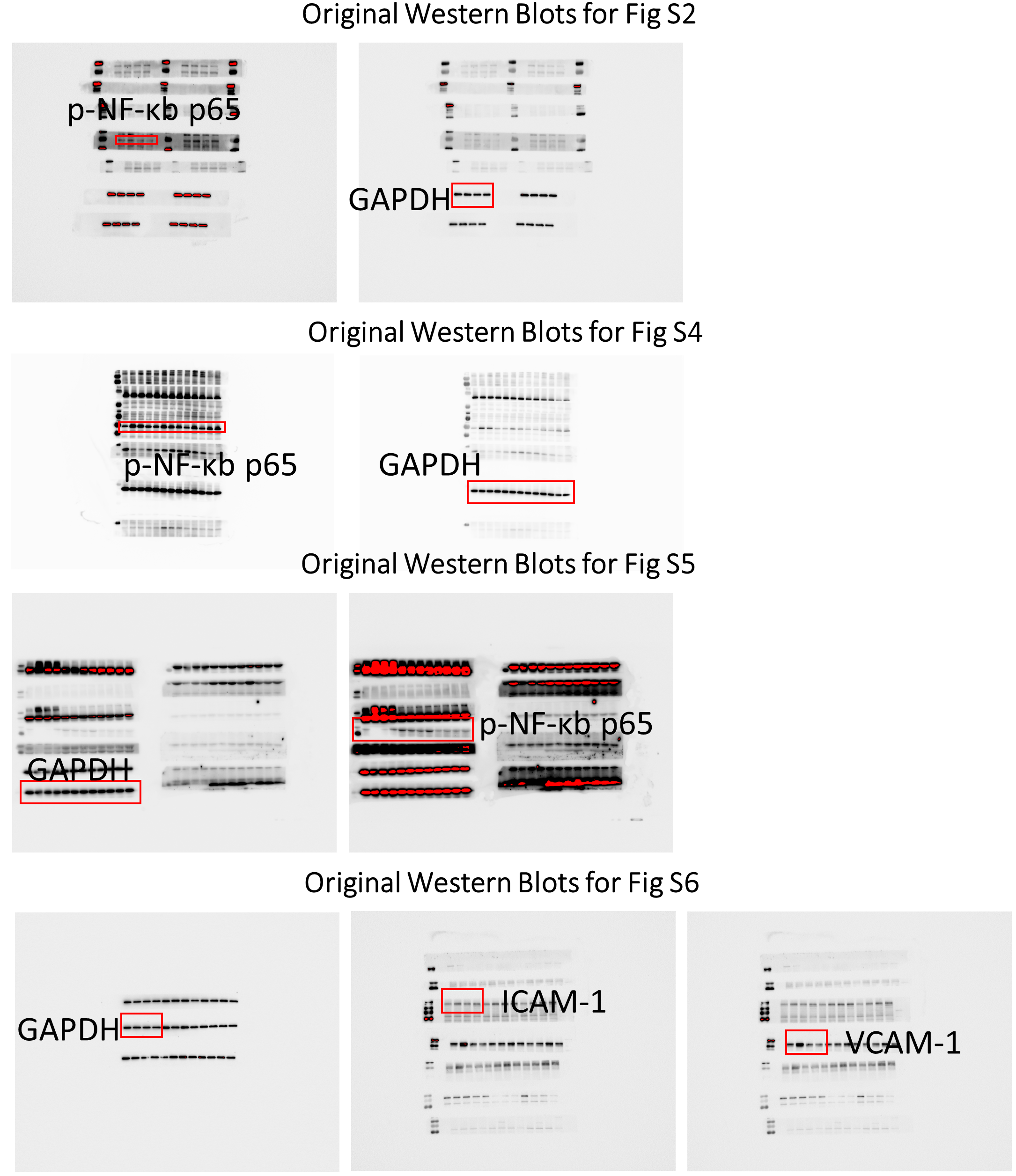

Supplement: Supplementary file 1 — Supporting Information [file 41420_2024_1921_MOESM1_ESM.docx]
